# Skeletal muscle effects of antisense oligonucleotides targeting glycogen synthase 1 in a mouse model of Pompe disease

**DOI:** 10.1002/ctm2.70314

**Published:** 2025-04-23

**Authors:** Lan Weiss, Michele Carrer, Alyaa Shmara, Angela Martin, Hong Yin, Pallabi Pal, Cheng Cheng, Lac Ta, Victoria Boock, Yasamin Fazeli, Mindy Chang, Marvin Paguio, Jonathan Lee, Howard Yu, John Weiss, Tamar R Grossman, Nina Raben, Paymaan Jafar‐Nejad, Virginia Kimonis

**Affiliations:** ^1^ Division of Genetics and Genomic Medicine Department of Pediatrics University of California Irvine California USA; ^2^ Ionis Pharmaceuticals, Inc. Carlsbad California USA; ^3^ Department of Neurology University of California Irvine California USA; ^4^ Cell and Developmental Biology Center, National Heart, Lung, and Blood Institute National Institutes of Health Bethesda Maryland USA; ^5^ Department of Pathology University of California Irvine CA USA; ^6^ Present address: Tamar R Grossman, La Jolla Labs, El Cajon, CA 92020; ^7^ Present address: Nina Raben, M6P Therapeutics, St. Louis, Missouri

**Keywords:** antisense oligonucleotides (ASOs), Enzyme replacement therapy (ERT), *Gaa^‐/‐^* mouse model, glycogen synthase 1 (GYS1), Pompe disease, skeletal muscle

## Abstract

Pompe disease (PD) is a progressive myopathy caused by the aberrant accumulation of glycogen in skeletal and cardiac muscle resulting from the deficiency of the enzyme acid alpha‐glucosidase (GAA). Administration of recombinant human GAA as enzyme replacement therapy (ERT) works well in alleviating the cardiac manifestations of PD but loses sustained benefit in ameliorating the skeletal muscle pathology. The limited efficacy of ERT in skeletal muscle is partially attributable to its inability to curb the accumulation of new glycogen produced by the muscle enzyme glycogen synthase 1 (GYS1). Substrate reduction therapies aimed at knocking down GYS1 expression represent a promising avenue to improve Pompe myopathy. However, finding specific inhibitors for GYS1 is challenging given the presence of the highly homologous GYS2 in the liver. Antisense oligonucleotides (ASOs) are chemically modified oligomers that hybridise to their complementary target RNA to induce their degradation with exquisite specificity. In the present study, we show that ASO‐mediated *Gys1* knockdown in the *Gaa^−/−^* mouse model of PD led to a robust reduction in glycogen accumulation in skeletal muscle. In addition, combining *Gys1* ASO with ERT slightly further reduced glycogen content in muscle, eliminated autophagic buildup and lysosomal dysfunction, and improved motor function in *Gaa^−/−^* mice. Our results provide a strong foundation for validation of the use of *Gys1* ASO, alone or in combination with ERT, as a therapy for PD. We propose that early administration of *Gys1* ASO in combination with ERT may be the key to preventative treatment options in PD.

**Key points:**

- Antisense oligonucleotide (ASO) treatment in a mouse model of Pompe disease achieves robust knockdown of glycogen synthase (GYS1).
- ASO treatment reduces glycogen content in skeletal muscle.
- Combination of ASO and enzyme replacement therapy (ERT) further improves motor performance compared to ASO alone in a mouse model of Pompe disease.

## Graphical abstract

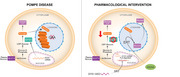

## 1 INTRODUCTION

Glycogen storage disease type II, also called Pompe disease (PD) is an autosomal recessive disorder affecting between 1 in 17 000 and 1 in 40 000 individuals.^1^ PD is caused by the excessive accumulation of glycogen in tissues resulting from inactivating mutations in the *GAA* gene, which encodes acid alpha‐glucosidase, an enzyme responsible for the lysosomal degradation of glycogen. The pathological accumulation of glycogen affects many tissues, but it is by far the most impactful in the heart and skeletal muscle of Pompe patients. Indeed, the lack of GAA enzyme activity, or extremely low levels (1%–2% of normal), are associated with fatal infantile cardiomyopathy, profound weakness, and skeletal muscle pathology, whereas 10%–20% of normal GAA levels lead to skeletal myopathy with childhood or adult onset.^2^, ^3^ To understand the role of GAA in Pompe disease etiology, a mouse model has been generated by targeted deletion of the murine *Gaa* gene. The *Gaa* knockout (*Gaa* ^−/−^) mouse recapitulates Pompe disease manifestations, including the accumulation of glycogen in skeletal and cardiac muscle, the autophagy defects, and the consequent myopathy.^4^

The standard of care for Pompe patients is enzyme replacement therapy (ERT) using recombinant human (rh) GAA (Lumizyme®, or Myozyme®, Sanofi Aventis US LCC), which became commercially available in 2006. ERT has shown positive results in improving the myopathy and cardiomyopathy associated with PD, and therefore has been used to help stabilise or partially improve patient outcomes.^5^, ^6^, ^7^ However, ERT has limited efficacy in the skeletal muscle, as many patients continue to develop progressive muscle weakness despite receiving rhGAA enzyme.^8^, ^9^, ^10^ Multiple factors contribute to the poor efficacy of ERT in the skeletal muscle of Pompe patients, particularly when significant disease pathology is present. Some of the limitations affecting ERT are associated with the complexity of delivering a relatively large protein to the muscle tissue. Indeed, when administered via intravenous infusion, the recombinant GAA enzyme circulates to the liver, which acts as a major “sink” and precludes more than 80% of the administered protein from reaching the skeletal muscle.^11^ Furthermore, ERT relies on the cation‐independent mannose‐6‐phosphate receptor (CI‐MPR), which is expressed at low levels on the membrane of skeletal muscle cells.^7^ In addition, dysregulation of autophagy, and the consequent impairment of the autophagosome‐lysosome fusion in Pompe myofibres, further contribute to the reduced efficacy of ERT. Ultimately, the failure of autophagy leads to inefficient trafficking of the therapeutic enzyme to the lysosome, limiting its ability to clear the glycogen.^12^ This phenomenon is especially evident in type II muscle fibres, which are particularly refractory to ERT.^10^ Previous studies performed in mice found upregulated protein levels of enzymes involved in glucose uptake and cytoplasmic glycogen synthesis in skeletal muscle from mice with Pompe disease, including glycogenin (GYG1), glycogen synthase (GYS1), glucose transporter 4 (GLUT4), glycogen branching enzyme 1 (GBE1), and UDP‐glucose pyrophosphorylase (UGP2) suggesting a positive feedforward loop for cellular glycogen accumulation.^13^, ^14^ Administration of rhGAA to the Pompe mice normalised not only muscle glycogen levels but also glucose‐6‐phosphate (G6P), hexokinase, glycogen synthase, and glycogenin levels.^14^ Thus, it would appear that correction of one aspect of the glycogen metabolic pathway (lysosomal glycogen) results in the correction of multiple other components. The elevated levels of cytoplasmic glycogen biosynthetic enzymes would appear to exacerbate the glycogen storage problem since rhGAA only has significant activity in the acidic lysosomal environment.^14^ Studies in patient biopsies however found that extralysosomal glycogen accumulation in muscle biopsies from late‐onset patients was not cleared by ERT, indicating the importance of targeting lysosomes with their acidic pH environment.^15^

Glycogen synthase (GYS) enzymes catalyse the addition of glucose residues to the growing glycogen molecules by formation of α‐1,4‐glucosidic linkages. Two paralogous GYS genes, GYS1 and GYS2, exist in mice and humans. GYS2 expression is restricted to the liver, where it plays an essential role in the maintenance of proper body glucose homeostasis.^16^ GYS1 has a broader expression profile, but it is particularly abundant in skeletal muscle and heart.^16^ Decreasing GYS1 expression represents a potential therapeutic approach for PD, based on the principle of substrate reduction. Indeed, limiting the synthesis of new glycogen would result in attenuated glycogen accumulation in the muscle tissue. Genetic ablation of murine *Gys1* provided proof of principle validation for substrate reduction therapy (SRT) in vivo. Knocking down *Gys1* led to restoration of muscle functionality in the *Gaa^−/−^* mouse model.^17^ Similarly, intramuscular injection of recombinant adeno‐associated virus‐1 (AAV‐1) vector expressing short hairpin ribonucleic acid (shRNA) targeted to *Gys1* has been shown to reduce glycogen accumulation in the gastrocnemius of newborn *Gaa^−/−^* mice.^18^ Altogether the preclinical evidence suggests that reducing GYS1 can be a potential therapeutic approach in Pompe disease, either alone, or in combination with ERT. However, GYS1 and GYS2 show a high degree of homology, which poses a complex challenge in the identification of safe, isoform‐specific inhibitors.^19^ It is important to note that no approved small molecule inhibitor is clinically available for the treatment of Pompe disease.

Antisense oligonucleotides (ASOs) are short synthetic nucleic acids that hybridise to complementary target RNAs in a sequence‐specific manner using Watson‐Crick base pairing. By doing so, ASOs can influence RNA processing and regulate protein expression via multiple mechanisms, including RNase H1‐mediated mRNA degradation, and splicing modulation.^20^, ^21^, ^22^, ^23^, ^24^ Over the past two decades, ASOs have emerged as both powerful research tools and therapeutic molecules.^25^, ^26^, ^27^, ^28^, ^29^ The intrinsic properties of ASOs, that is, binding target mRNAs via complementary base pairing to induce RNase H1‐mediated mRNA degradation, make them the ideal pharmacological tool to reduce muscle GYS1 expression with exquisite specificity, particularly since GYS1 and GYS2 show such a high degree of homology. Here, we show that systemic treatment with a phosphorothioate (PS) 2′‐constrained ethyl (cEt)‐modified gapmer ASO targeting murine *Gys1* not only reversed the excessive glycogen accumulation, but also corrected the autophagic defects present in the skeletal muscle of *Gaa^−/−^* mice. The ASO‐mediated reduction of GYS1 protein, especially when combined with ERT, led to the restoration of muscle function in Pompe mice, thus underscoring the in vivo potential of our approach.

## 2 RESULTS

### 2.1 Progressive glycogen accumulation and muscle weakness in the *Gaa^−/−^* mouse model

**FIGURE 1:**
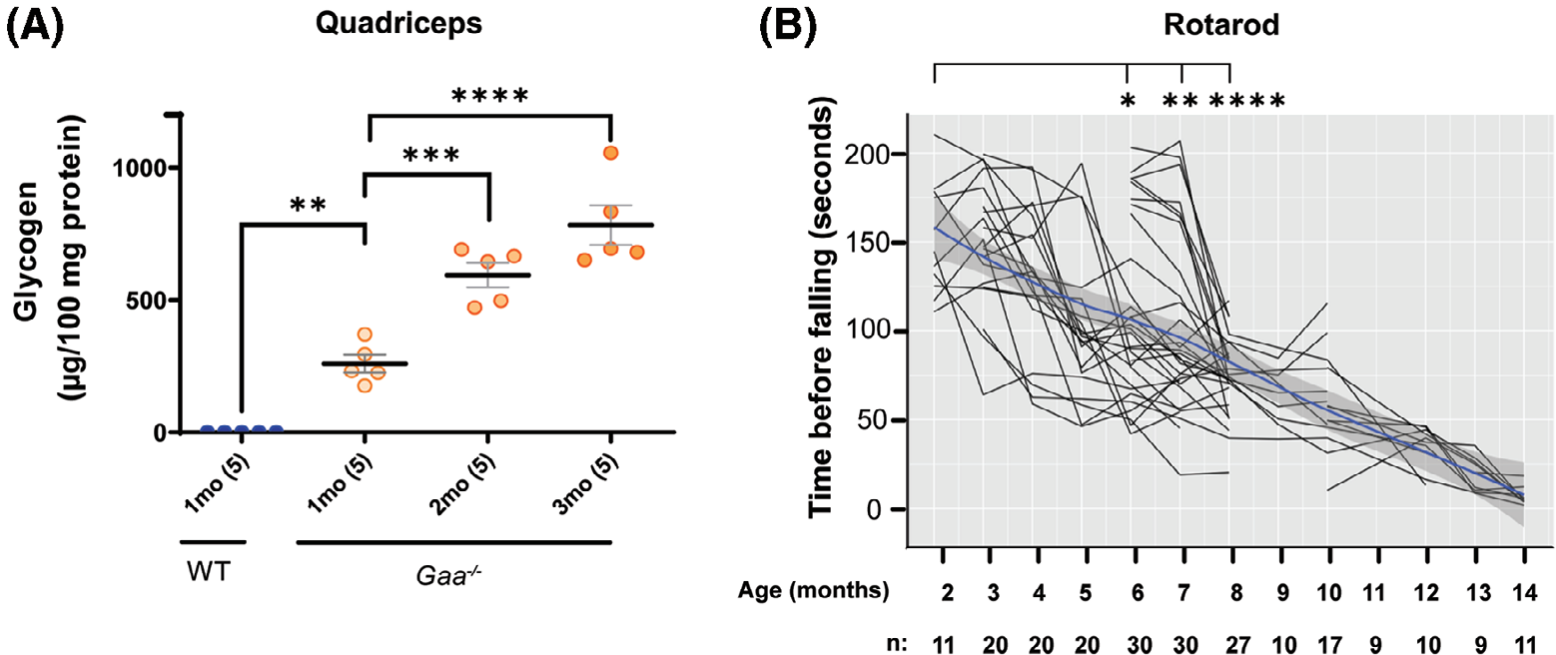
*Gaa^−^^/^^–^* mice show progressive accumulation of glycogen in skeletal muscle followed by deterioration of muscle function. (A) Glycogen content in quadriceps from *Gaa^−/−^* mice at 1, 2, and 3 months (mo) of age compared to 1‐month‐old wild‐type (WT) C57BL/6 mice. *n* = 5 per group, as indicated in parenthesis on the *x*‐axis. (B) Graph illustrates the time that the mice spent on an accelerating rotarod before falling, to assess muscle strength as the animals age from 2 to 14 months. The mean decline in motor function is represented by the blue line within the shaded grey area, which indicates the SEM range. The number of mice in each measurement group is indicated at the bottom of the graph (*n*). Statistical analysis in A and B were performed using one‐way ANOVA in GraphPad Prism software. *p < .05, **p < .01, ***p < .005, ****p < .001.

We utilised the *Gaa^−/−^* mouse model to study the effects of lowering *Gys1* expression on Pompe myopathy in vivo. The *Gaa^−/−^* mouse was generated and studied by Raben et al. (1998).^4^ To inform the design of appropriate prevention and reversal treatment paradigms, we characterised the progressive accumulation of glycogen in the skeletal and cardiac muscle of the *Gaa^−/−^* mice from our colony. We utilised two methods to measure glycogen content in the tissues: a colorimetric biochemical assay, and Periodic acid‐Schiff (PAS) histochemical staining.^4^ We observed higher amounts of glycogen in the skeletal muscle of *Gaa* ^−/−^ mice compared to wild‐type mice (Figure 1A). Remarkably, the glycogen levels in quadriceps muscle were already elevated in 1‐month‐old *Gaa* ^−/–^ mice, suggesting that the mutant mice start to accumulate glycogen in skeletal muscle at an early age.

The accumulation of glycogen increased progressively over time, from 260 µg (95% confidence interval (C.I.): 167–353 µg), to 595 µg (95% C.I.: 467– 722), and 784 µg (95% C.I.: 575–992) glycogen per 100 mg tissue in quadriceps muscle homogenate from 1‐, 2‐, and 3‐month‐old *Gaa* ^−/−^ mice, respectively (Figure 1A). Similar to previous reports,^4^, ^30^, ^31^ the excessive glycogen accumulation in the skeletal muscle was associated with a progressive decline in motor function in aging *Gaa* ^−/−^ mice, as assessed by the rotarod test (Figure 1B).

### 2.2 Different levels of ASO‐mediated *Gys1* knockdown lead to the proportional amelioration of aberrant glycogen accumulation in the skeletal muscle of young *Gaa* ^−/−^ mice

Through in vitro and in vivo screening, we identified two ASOs targeting mouse *Gys1* that were potent and well tolerated in mice (Figures S1 and S2). When dosed systemically to wild‐type mice via subcutaneous injection for 6 weeks, *Gys1* ASO#1 and ASO#2 resulted in the efficient, dose‐dependent knockdown of murine *Gys1* mRNA. ASO#1 had an ED_50_ of 45.2 mg/kg (95% C.I.: 32.3–63.2 mg/kg) in the quadriceps muscle, whereas ASO#2 was more potent, with an ED_50_ of 24.2 mg/kg (95% C.I.: 22.4–26.1 mg/kg) (Figure S1A). Consistently, *Gys1* ASO#2 achieved a robust reduction of GYS1 protein in skeletal muscle of wild‐type and *Gaa* ^−/−^ mice (Figure S1B–D).

**FIGURE 2:**
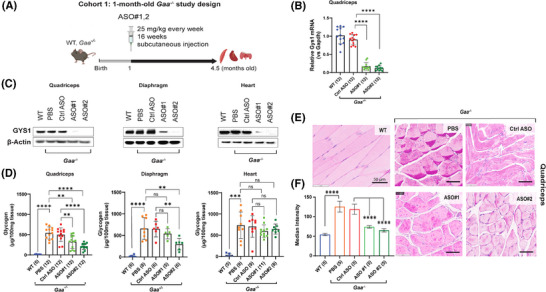
Early treatment of *Gaa^−^^/^^−^* mice with *Gys1* ASOs lowers target mRNA and protein expression, leading to reduced glycogen accumulation in skeletal muscle. (A) Schematic illustration of the early treatment paradigm with *Gys1* ASOs in *Gaa^−/−^* mice from 1 to 4.5 months of age. WT: wild type. (B) qPCR analysis of *Gys1* mRNA in quadriceps muscle. (C) Representative images of Western blot analysis of GYS1 protein in quadriceps, diaphragm, and heart (*n* = 5–7 per group). (D) Glycogen content measured using a biochemical assay in quadriceps muscle, diaphragm, and heart from *Gaa^−/−^* mice dosed with either PBS, control ASO (Ctrl ASO), or two different *Gys1* ASOs. The number of samples in each group is listed in parentheses on the *x*‐axis. (E) Periodic acid‐Schiff (PAS) staining of histological sections of quadriceps muscle. Scale bars: 50 µm. (F) PAS median intensities of quadriceps muscle fibres in controls and treated groups. Statistical analysis was performed using one‐way ANOVA in GraphPad Prism software. ***p < .005, ****p < .0001.

To determine if ASO‐mediated *Gys1* knockdown attenuates the pathological accumulation of glycogen in vivo, we dosed young *Gaa* ^−/−^ mice with the newly identified *Gys1* ASO#1 and ASO#2. A starting age of 1 month was selected because at this time the *Gaa* ^−/−^ mice have not developed severe myopathy yet, and the glycogen is only starting to accumulate in the skeletal muscle (Figure 1A). The 1‐month‐old *Gaa* ^−/–^ mice (Cohort 1) were dosed via subcutaneous injection with 25 mg/kg *Gys1* or control ASO, once a week for 16 weeks (Figure 2A). Compared to control ASO, *Gys1* ASO#1 and ASO#2 resulted in 83.64% (95% C.I.: 77.40–88.58) and 88.58% (95% C.I.: 83.61–92.06) *Gys1* mRNA knockdown, respectively in quadriceps muscle (Figure 2B, Table S1). Corroborating the reduction in mRNA expression, compared to control ASO, ASO#2 administration decreased GYS1 protein, measured by Western blot, in key Pompe tissues of *Gaa* ^−/−^ mice by 100%, 98%, and 70% in quadriceps, diaphragm, and heart, respectively. (Figure 2C, Table S1).

To evaluate the impact of *Gys1* knockdown on excessive glycogen accumulation in the muscle of the Pompe mice, we measured glycogen content by a colorimetric biochemical assay in the muscle of the 20‐week‐old *Gaa* ^−/−^ mice following 16 weeks of *Gys1* ASO treatment. Compared to the control ASO, treatment with ASO#1 led to a 41.13% reduction in glycogen content in the quadriceps (322 µg/100 mg tissue vs. 501 µg/100 mg tissue) and a 15.12% reduction in the diaphragm (539 µg/100 mg tissue vs. 648 µg/100 mg tissue). In contrast, ASO#2 resulted in a more substantial decrease, with glycogen levels falling by 58.47% in the quadriceps (212 µg/100 mg tissue vs. 501 µg/100 mg tissue) and by 57.07% in the diaphragm (296 µg/100 mg tissue vs. 648 µg/100 mg tissue) (Figure 2D, Table S1). In the heart, we observed variable degree of reduction of GYS1 protein levels, on average the impact of both ASOs was more pronounced in quadriceps and diaphragm (Figure 2C, Table S1). The control ASO had no effect on *Gys1* expression and glycogen content (Figure 2B–F).

In line with the biochemical measurements, glycogen content assessed in histological sections using PAS staining confirmed that *Gys1* ASOs reduced aberrant glycogen accumulation in the quadriceps muscle of *Gaa^−/−^* mice (Figure 2E). Quantitative analysis of PAS‐stained images using Imaris revealed a median intensity of 119 (95% C.I.: 103–135) in 4.5‐month‐old *Gaa^−/−^* mice treated with control ASO, 74 (95% C.I.: 70–78) in mice treated with ASO#1, and 65 (95% C.I.: 60–70) in mice treated with ASO#2 (Figure 2E and F). In view of the enhanced efficacy in the early‐treatment setting, we selected ASO#2 to further investigate if *Gys1* ASO treatment could revert the Pompe pathology in older *Gaa^−/−^* mice.

### 2.3 ASO‐mediated *Gys1* reduction partially reverses glycogen accumulation in the skeletal muscle of older *Gaa^−/−^* mice

**FIGURE 3:**
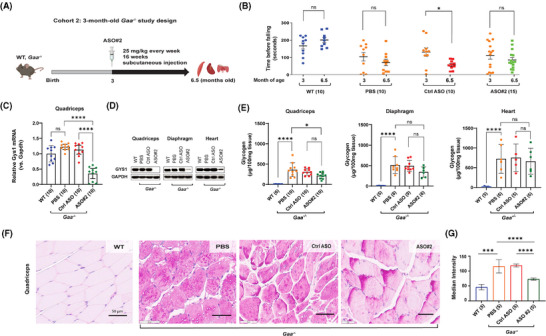
Therapeutic treatment with *Gys1* ASO in *Gaa^−^^/^^–^* mice partially reverses glycogen accumulation in skeletal muscle and preserves muscle strength. (A) Schematic illustration of the reversal treatment paradigm with *Gys1* ASO#2 in *Gaa^−/−^* mice from 3 to 6.5 months of age. WT: wild type. (B) Graph illustrates the time that mice spent on an accelerating rotarod before falling, to assess muscle strength. (C) qPCR analysis of *Gys1* mRNA in quadriceps muscle. The number of samples in each group is listed in parentheses on the *x*‐axis. (D) Representative images of Western blot analysis of GYS1 protein in quadriceps, diaphragm, and heart (*n* = 5 per group). GAPDH was used as loading control. (E) Glycogen content measured using a biochemical assay in quadriceps muscle, diaphragm and heart from *Gaa^−/−^* mice dosed with either PBS, a control ASO (Ctrl ASO), or *Gys1* ASO. The number of samples in each group is listed in parentheses on the *x*‐axis. (F) Representative images from PAS staining of histological sections of quadriceps muscle. Scale bars: 50 µm. (G) PAS median intensities of quadriceps muscle fibres in controls and treated groups. Statistical analysis was performed using unpaired *t*‐test (Figure 3B) or one‐way ANOVA in GraphPad Prism software. *p < .05, ***p < .005, ****p < .001, ns: not significant.

The typical patient with late onset Pompe disease presents in the clinic is in their 30s–40s, when the disease has already progressed. To assess if therapeutic treatment with *Gys1* ASO could reverse the glycogen accumulation and myopathy in aged model, we dosed *Gaa^−/−^* mice with *Gys1* ASO#2 starting at 3 months of age (Cohort 2) (Figure 3A). The *Gys1* ASO#2 was administered by weekly subcutaneous injections at 25 mg/kg for 16 weeks (Figure 3A).

The rotarod motor performance assay showed that the ASO#2‐mediated knockdown of *Gys1* expression did not significantly slow down the deterioration of motor function in Pompe mice compared to the control ASO. However, minor improvements were observed in 5 out of 10 ASO#2‐treated animals, whereas only 1 animal in the PBS group and none in the control ASO group showed improvement (Figure 3B). After completing the treatment, we sacrificed the animals at 6.5 months of age to study the quadriceps muscle, diaphragm, and heart tissues.

In the quadriceps muscle, we found an 83.34% (95% C.I.: 89.65–73.21) knockdown of mouse *Gys1* mRNA and complete depletion of GYS1 protein compared to control ASO (Figure 3C and D, Table S1). Similarly, GYS1 protein was reduced by 80% in diaphragm and 70% in heart compared to mice treated with control ASO (Figure 3D, Table S1). Interestingly, the expression of *Gys1* mRNA and GYS1 protein in quadriceps muscle of 6.5‐month‐old Pompe mice was not significantly different from that of the wild‐type mice (Figure 3C and D). This may be because the key difference between Pompe and wild‐type muscle GYS1 expression is not in total protein levels but in GYS1 phosphorylation, as reported by Ullman et al. (2024).^32^

A biochemical assay demonstrated that the ASO#2‐mediated knockdown of *Gys1* expression was associated with a trend toward reduced glycogen accumulation in the quadriceps (32.66% reduction; 214 µg vs. 307 µg/100 mg tissue, not significant by one‐way ANOVA, *p* < .02 by unpaired t test) and the diaphragm (32.33% reduction; 343 µg vs. 492 µg/100 mg tissue, not significant with one‐way ANOVA, *p* < .05 with unpaired t test) of *Gaa^−/−^* mice, compared to the control ASO. In contrast, glycogen accumulation in the heart was 660.5 µg/100 mg in ASO#2‐treated mice versus 752.5 µg/100 mg in control ASO‐treated mice (*p* = .88), indicating no statistically significant difference between the groups (Figure 3E, Table S1). The lower GYS1 protein knockdown efficiency in the heart (70%) compared to the quadriceps (98%) and diaphragm (85%) likely accounts for the reduced impact on glycogen reduction in cardiac tissue. Furthermore, quantitative analysis of histological sections of the quadriceps muscle using PAS staining confirmed an improvement of the aberrant glycogen accumulation following treatment with *Gys1* ASO#2. The median staining intensity was 119 (95% C.I.: 113–125) in 6.5‐month‐old *Gaa^−/−^* mice treated with control ASO, and 73 (95% C.I.: 69–77) in mice treated with ASO#2 (Figure 3F). To minimise technical variability in the PAS staining data, all samples from every cohort were processed simultaneously. Notably, a similar PAS staining mean intensity of 119 was observed in the quadriceps of Pompe mice treated with control ASO in both cohort 1 (4.5 months old, Figure 2E and F) and cohort 2 (6.5 months old, Figure 3E and F), despite differences in glycogen content: 501 µg/100 mg tissue in cohort 1 versus 307 µg/100 mg tissue in cohort 2. This discrepancy underscores the semi‐quantitative nature of PAS staining.

### 2.4 ASO‐mediated *Gys1* knockdown in combination with enzyme replacement therapy further reverses Pompe pathology, preserving motor function in older *Gaa^−/−^* mice

**FIGURE 4:**
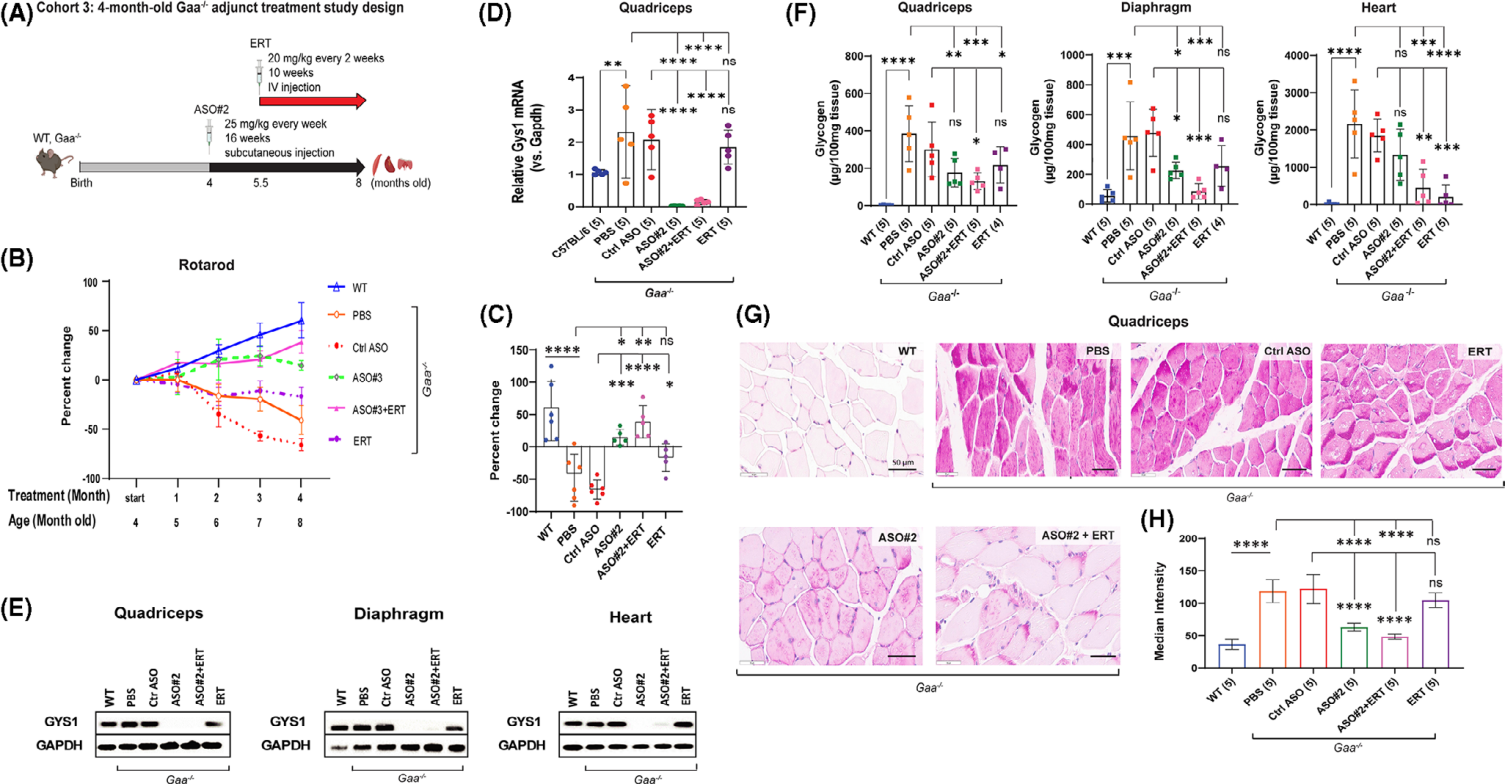
Combination treatment with *Gys1* ASO and ERT reduces glycogen accumulation and improves motor function in *Gaa^−^^/^^−^* mice. (A) Schematic illustration of the study where 4‐month‐old *Gaa^−/−^* mice were dosed with *Gys1* ASO#2 and ERT in combination. WT: wild type. (B) Graph shows the results of a rotarod test to assess muscle strength. The time that the mice spent on a rotating rod was recorded monthly over the 3 months of treatment and expressed as per cent change versus baseline measurements (collected before the start of dosing) for each group; *n* = 5 per group. (C) Per cent change of rotarod data at the final time point. Statistical analysis in B and C were performed by two‐way ANOVA using multiple comparisons in GraphPad Prism software. ****p < .001 between the *Gaa^−/−^* mice that received control ASO and *Gys1* ASO#2 or *Gys1* ASO#2+ERT. (D) qPCR analysis of *Gys1* mRNA in quadriceps muscle. The number of samples in each group is listed in parentheses on the *x*‐axis. (E) Representative images of Western blot analysis of GYS1 protein in quadriceps muscle (*n* = 5), diaphragm (*n* = 3), and heart (*n* = 5 per group). GAPDH was used as loading control. (F) Glycogen content measured using a biochemical assay in quadriceps muscle, diaphragm and heart from wild‐type or *Gaa^−/−^* mice dosed with either PBS, *Gys1* ASO#2, *Gys1* ASO#2+ERT, or ERT alone (*n* = 5 per group). (G) Representative images from PAS staining of histological sections of quadriceps muscle. Scale bars: 50 µm. (H) PAS median intensities of quadriceps muscle fibres in controls and treated groups, data are the mean ± SD from 5 images each group. Statistical analysis in D, F and H were performed using one‐way ANOVA. *p < .05, **p < .01, ***p < .005, ns: not significant.

Substrate reduction therapy (SRT) using ASO to remove the source of new glycogen synthesised via GYS1 showed benefit for skeletal muscle, with variable effect on the heart in both 4.5‐ and 6.5‐month‐old *Gaa^−/−^* mice. We reasoned that combining *Gys1* ASO, which blocks the synthesis of new glycogen, with GAA enzyme replacement therapy (ERT) to simultaneously remove preexisting glycogen in the skeletal muscle and heart of older Pompe mice, could provide additive efficacy in muscle and especially the heart. Therefore, we dosed 4‐month‐old *Gaa^−/−^* mice with *Gys1* ASO#2 at 25 mg/kg by weekly subcutaneous injections for 6 weeks, followed by a 10‐week‐long co‐administration phase where we combined ASO#2 and ERT, in the form of recombinant human alpha‐acid glucosidase (rhGAA) enzyme (Cohort 3) (Figure 4A). rhGAA was dosed intravenously every 2 weeks. Alternatively, Gaa^−/−^ mice were dosed with either ASO#2 alone or ERT alone (Figure 4A). Three groups—wild‐type, PBS, and control ASO‐treated *Gaa^−/−^* mice—were included in the study as controls, with the latter being used as the best comparator for the *Gys1* ASO#2‐treated *Gaa^−/−^* mice.

The ASO#2+ERT combination treatment led to a steady increase in rotarod performance over the 4‐month treatment period, with gains of 18%, 16%, 21%, and 39%, respectively compared to control ASO after 1, 2, 3, and 4 months of treatment. In contrast, ASO#2 alone increased rotarod performance during the first 3 months by 3%, 20%, and 25%, and 15% gain compared to control ASO. ERT alone was unable to prevent the functional decline in aging Pompe mice, resulting in a downward change in rotarod performance by –4%, –17%, –11%, and –17% after 1, 2, 3, and 4 months of treatment, respectively. The control ASO group in contrast showed significant declines of –7%, –35%, –57%, and –66% over the same time period (Figure 4B). The histogram in Figure 4C clearly highlights the effects of ASO#2 alone, ASO#2+ERT and ERT alone treatment on rotarod performance at the final time point.

At the end of the study, the 8‐month‐old mice were sacrificed for further biochemical and IHC analysis of the muscle tissue. Treatment with ASO#2, either alone or in combination with ERT, resulted in a 93%–99% reduction in mRNA expression and complete ablation of GYS1 protein in the quadriceps (Figure 4D and E). Similarly, minimal residual GYS1 protein, with 85–90% reduction was detected in the diaphragm and heart with the same treatment groups. As expected, treatment with ERT alone reduced GYS1 protein levels in the quadriceps and diaphragm of *Gaa^−/−^* mice by 25% compared to control ASO treated mice. However, ERT had no effect on the heart, where GYS1 protein expression remained similar to control ASO treated *Gaa^−/−^* mice (Figure 4E, Table S1).

Western blot analysis revealed pronounced GYS1 protein expression in quadriceps, diaphragm and heart of *Gaa^−/−^* mice treated with PBS, approximately 20%–30% higher than in wild‐type mice, while *Gys1* mRNA levels showed more than a two‐fold increase in quadriceps (Figure 4D and E).

Importantly, the systemic administration of *Gys1* ASO#2, alone or in combination with ERT, was well tolerated in Pompe mice with no significant changes in body or organ weights, and no significant elevation in liver transaminases, blood urea nitrogen, or muscle creatine kinase in plasma compared to control groups (Figure S2).

Compared to control ASO, the biochemical glycogen assay revealed that ASO#2 alone did not result in a statistically significant reduction in quadriceps muscle glycogen content (177 µg vs 300.5 µg/100 mg tissue, *p* = .16). However, ASO#2 alone led to a significant 50.86% reduction in diaphragm glycogen levels (227 µg vs. 478 µg/100 mg tissue, *p* < .05) in *Gaa^−/−^* mice (Figure 4F, Table S1). The ASO#2+ERT combination had an even greater effect, reducing glycogen levels by 54.65% in quadriceps muscle (131 µg vs. 300.5 µg/100 mg tissue) and by 83.13% in the diaphragm (84 µg vs. 478 µg/100 mg tissue) (Figure 4F, Table S1).

Compared to control ASO, ERT alone had some minor, but not significant effect on glycogen accumulation in *Gaa^−/−^* mice, with quadriceps glycogen content at 217.5 µg vs. 300.5 µg/100 mg tissue (*p* = .48), and diaphragm glycogen at 256 µg vs. 478 µg/100 mg tissue (*p* = .07). ERT was more efficacious in the heart, leading to a 97.72% reduction in heart glycogen content compared to control ASO when dosed alone (213 µg vs. 1854 µg/100 mg tissue), and a 87.51% reduction when combined with *Gys1* ASO#2 (456 µg vs. 1854 µg/100 mg tissue), and a 34.55% reduction when ASO#2 was used alone (1335 µg vs. 1854 µg/100 mg tissue) (Figure 4F, Table S1).

PAS staining of quadriceps muscle histological sections corroborated the biochemical assay results, showing a marked reduction in glycogen content, particularly in the ASO#2 group (median intensity: 63, 95% C.I.: 56– 71) and the ASO#2+ERT group (median intensity: 48, 95% C.I.: 43–53), compared to the control ASO group (median intensity: 122, 95% C.I.: 94–150) and the ERT alone group (median intensity: 104.5, 95% C.I.: 90–119) (Figure 4G and H). Thus, the motor function improvement correlated well with the reduction in muscle glycogen content.

### 2.5 Effect of *Gys1* ASO and ERT on muscle regeneration in *Gaa^−/−^* mice

Centrally located nuclei are a hallmark feature observed in certain muscle disorders, particularly those involving muscle regeneration and repair indicating newly regenerated myofibres following damage.^33^, ^34^ Approximately 600 quadriceps myofibres from 3–5 mice per group (1–2 images/mouse) were analysed for centralised nuclei. In the 8‐month‐old *Gaa* ^−/−^ mice, we observed a significant increase in the percentage of myofibre with centralised nuclei in quadriceps muscle (1.5%, 95% CI: 0.3–3.3) compared with WT mice (1.1%, 95% CI: 21.4–60.9), confirming impaired muscle regeneration (Figure S3). Treatment with Gys1 ASO#2, either alone or in combination with ERT, or ERT led to alleviation these abnormalities. The percentage of myofibre with centralised nuclei in *Gaa^−/−^* mice treated with Gys1 ASO#2 was 20.7% (95% CI: 5.2–36.3), while the combined Gys1 ASO#2 + ERT treatment reduced it to 11.9% (95% CI: 1.3–22.4). ERT alone also provided some improvement, with a reduction to 18.7% (95% CI: 9.0–28.5), compared to the control ASO group, which exhibited a high percentage of centralised nuclei at 48.9% (95% CI: 31.4–66.5) (Figure S3). These results suggest that Gys1 ASO#2, with or without ERT, may enhance muscle regeneration and alleviate some of the pathological features associated with Pompe disease.

Taken together, our data indicates that blocking the synthesis of new glycogen while concomitantly enhancing its clearance is beneficial in treating both the skeletal muscle and cardiac abnormalities in Pompe disease (Figure 6).

### 2.6 ASO‐mediated *Gys1* knockdown with or without ERT, but not ERT alone, ameliorates the autophagic markers in the skeletal muscle of *Gaa^−/−^* mice

Defective autophagy is a major factor contributing to the muscle pathology in Pompe disease.^7^ Consistent with the highly integrated functions of lysosomes and autophagosomes, it has been shown that lysosomal dysfunction in Pompe disease leads to a block in autophagic flux and accumulation of autophagic debris, particularly in muscle tissue.^35^ It is important to note that the *Gaa^−/−^* mouse model faithfully recapitulates the autophagic defects described in tissues from Pompe patients.^30^

**FIGURE 5:**
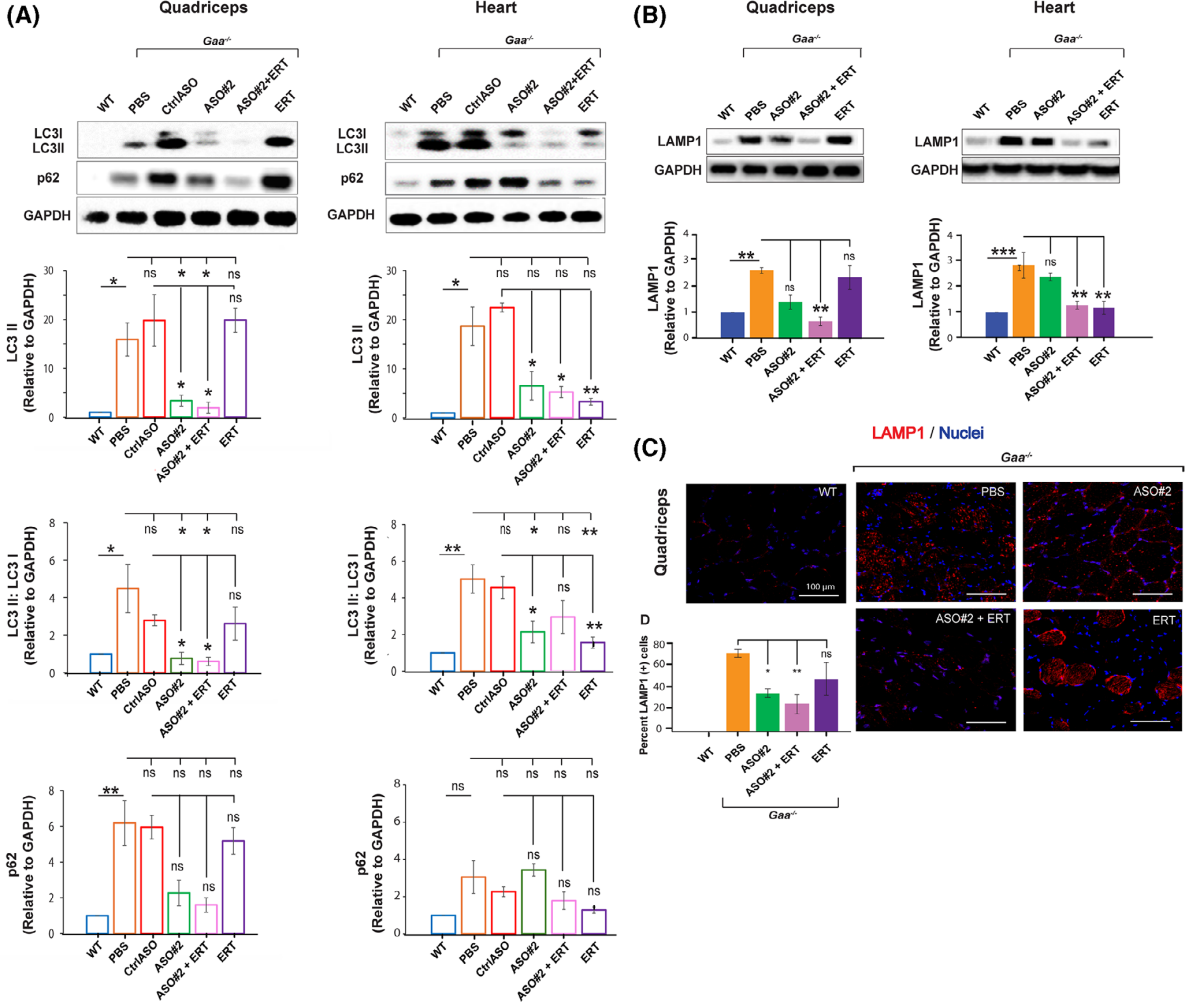
Normalisation of autophagic flux in skeletal muscle of Pompe mice after treatment with *Gys1* ASO, alone or in combination with ERT. (A) Western blot analysis of LC3‐II, LC3‐II/LC3‐I ratio, and p62 proteins in quadriceps muscle and heart from mice in cohort 3. GAPDH was used as loading control. WT: wild type. (B) Western blot analysis of LAMP1 expression. (C) Representative images of LAMP1 immunostaining in histological sections of quadriceps muscle from *Gaa^−/−^* mice treated with *Gys1* ASO#2, alone or in combination with ERT, scale bars: 100 µm. (D) Quantification of LAMP1‐positive cells as percentage of the total cell count in quadriceps muscle sections. LAMP1: lysosomal‐associated membrane protein 1. All experiments were conducted with *n* = 3–5 samples per group. The graphs depict the quantification of Western blots from three to five independent experiments. Statistical analysis was performed using one‐way ANOVA. *p < .05, **p < .01, ***p < .005, ns: not significant.

**FIGURE 6:**
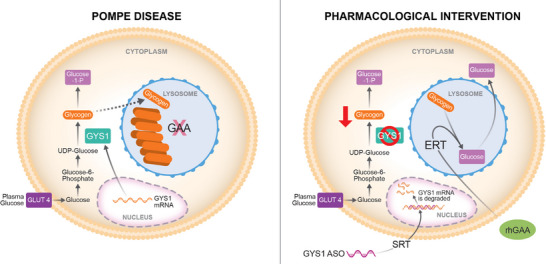
Model of pharmacological intervention in Pompe disease combining *GYS1* ASO‐based SRT and ERT. Proposed adjunct therapeutic approach for Pompe disease based on the reduction of the synthesis of new glycogen via *GYS1* ASO‐mediated SRT, combined with the ERT‐mediated clearance of preexisting glycogen that has accumulated in the lysosome because of GAA inactivation. Such combinatorial strategy could potentially result in greater benefit for Pompe patients, especially in skeletal muscle, where ERT alone is not remarkably effective. SRT: substrate reduction therapy; ERT: enzyme replacement therapy; GYS1: glycogen synthase 1; ASO: antisense oligonucleotide; GAA: glucosidase, alpha acid; rhGAA: recombinant human GAA.

Incomplete autophagic flux can be assessed by measuring the aberrant accumulation of LC3 and p62 proteins. Additionally, immunostaining of histological sections for lysosomal‐associated membrane protein 1 (LAMP1) provides a method to detect enlarged lysosomes.^36^, ^37^ We investigated the efficacy of *Gys1* ASO, alone or in combination with ERT (Cohort 3, Figure 4A), in ameliorating the defective autophagy in *Gaa^−/−^* mice. Western blot analysis confirmed accumulation of p62 and LC3‐II proteins in quadriceps muscle and heart of 8‐month‐old *Gaa^−/−^* mice treated with PBS (Figure 5A). ERT alone alleviated the autophagic defect in the heart but not significantly in the skeletal muscle, as indicated by the reduced levels of LC3‐II and LAMP1 proteins in cardiac tissue with no major effect in quadriceps muscle (Figure 5A and B), highlighting the tissue‐specific limitation of ERT. On the other hand, while *Gys1* ASO#2 contributed to a lesser extent, combination *Gys1* ASO#2 with ERT resulted an additive benefit, significantly enhancing the reduction of the autophagic LC3, LAMP1 markers in both skeletal and cardiac muscles (Figure 5A and B). In addition, immunostaining of histological sections confirmed that ASO#2+ERT administration reduced LAMP1 protein levels in quadriceps muscle of *Gaa^−/−^* mice (Figure 5B), suggesting that the combination therapy could rescue the autophagy pathway dysfunction in Pompe muscle after 4 months of treatment. The effect was more pronounced in the ASO#2+ERT group than in the *Gys1* ASO#2‐only group (Figure 5B). Our data indicates that the combination of *Gys1* ASO and ERT might provide therapeutic benefit by targeting various aspects of Pompe pathogenesis, namely glycogen accumulation and autophagy defects (Figure 6).

### 2.7 Systemic *Gys1* knockdown does not mitigate Pompe pathology in the central nervous system

Pompe disease is a complex multi‐systemic condition with growing evidence of central nervous system (CNS) involvement.^38^ Indeed, we found remarkable abnormalities in the spinal cord of *Gaa^−/−^* mice at 6.5 months of age (Figure S4). The pathologic changes were visible in histological sections of the spinal cord, where Nissl staining (toluidine blue) revealed the presence of swollen cell bodies, activated glial cells, and central nucleated cells. Indeed, we observed a statistically significant increase in number of neurons with eccentric nuclei in the spinal cord of *Gaa^−/−^* mice compared to WT controls (*p* < .0001, Fisher's Exact Tests, 30–40 neurons per group; Figure S3, data not shown). Subcutaneous injection of either control ASO or *Gys1* ASO#2 did not ameliorate the CNS phenotype of *Gaa^−/−^* mice (Figure S4, data not shown). These results are consistent with the inability of systemically dosed unconjugated ASOs to cross the blood‐brain barrier.^39^, ^40^, ^41^

## 3 DISCUSSION

Enzyme replacement therapy, currently the standard of care for patients with PD, has been shown to be beneficial, but its efficacy is hindered by some limitations, especially in treating the skeletal muscle manifestations of PD. Early administration of ERT improves cardiac hypertrophy and motor development, extending the chances of survival in patients with infantile‐onset disease (IOPD).^6^, ^42^ Patients with the late‐onset form (LOPD) have early improvement and stabilisation of their pulmonary and motor function, however after reaching a plateau there is continued decline in their motor function such as the 6 Minute Walk Test.^8^, ^9^ Progressive muscle weakness thus continues to be a problem for long‐term survivors of PD.^5^, ^6^ An explanation is provided by studies that have shown that the delivery of the therapeutic enzyme to skeletal muscle is insufficient to reduce the glycogen content and eliminate the autophagic buildup.^6^

Proof of principle for the utility of GYS1 inhibition for the treatment of Pompe disease was provided by Douillard‐Guilloux et al. (2008) who demonstrated that intramuscular injection of AAV‐1 expressing shRNA targeted to *Gys1* reduces glycogen accumulation in the gastrocnemius of newborn *Gaa^−/−^* mice.^19^ The same research group also analysed the effect of a complete genetic ablation of glycogen synthesis in a new *Gaa/Gys1* double knock‐out (KO) mouse model.^17^ The *Gaa/Gys1* KO mouse exhibited a profound reduction in cardiac and skeletal muscle glycogen, a significant decrease in lysosomal swelling and autophagic buildup, and complete correction of cardiomegaly.^17^ Muscle atrophy, observed in 11‐month‐old single Gaa KO mice, was less pronounced in the *Gaa/Gys1* double KO mice, resulting in improved exercise capacity.^17^ This data demonstrates that long‐term suppression of muscle glycogen synthesis is well tolerated and leads to a significant improvement in Pompe pathology. However, no approved small molecule inhibitors of muscle GYS1 are currently clinically available, underscoring the challenges of finding specific compounds that can preserve the activity of the homologous hepatic GYS2 enzyme. Recently, a study using a new adjunct therapy based on the enzyme and chaperone ATB200/AT222 combination showed enhanced efficacy over the currently approved ERT in *Gaa^−/−^* mice. ATB200/AT222 was able to normalise muscle glycogen content and reduced the levels of lysosomal and autophagosomal markers in the muscle of *Gaa^−/−^* mice to wild‐type levels.^7^, ^18^ Clinical trials of AT2221/ATB200 have been completed and this treatment was recently approved thus offering an alternative effective therapeutic option for Pompe disease patients. Approval however was based on noninferiority to the standard of care with lumizyme, and long‐term studies on its efficacy on muscle glycogen, autophagic buildup and muscle weakness is pending.

ASO technology has emerged as a highly specific therapeutic option for a variety of neuromuscular diseases. ASOs stand out from other technologies by offering precise targeting, reversible effects, and enhanced safety. They avoid risks commonly associated with immunogenicity, limited loading capacity, and off‐target effects observed in other approaches. RNA targeting ASOs have been approved for commercial use for several rare genetic diseases and clinical trials are in progress for rare and common diseases.^43^, ^44^, ^45^, ^46^, ^47^ ASOs that modulate exon splicing of the dystrophin (DMD) gene can reduce muscle degeneration in Duchenne's muscular dystrophy.^11^, ^48^, ^49^, ^50^, ^51^, ^52^ An ASO that disrupts spliceosome action on exon 7 of the survival motor neuron 1 (SMN1) gene and restores the gene to functioning length has been approved for the treatment of spinal muscular atrophy.^53^, ^54^ Recently, an ASO with RNase H1 mechanism has been approved for amyotrophic lateral sclerosis (ALS) patients with SOD1 mutations.^42^, ^43^ Several studies have attempted to use ASOs to mitigate the disease outcomes by improving or restoring normal GAA synthesis.^6^ Van der Wal et al. (2017) studied the common IVS132‐13T > G GAA mutation and located sequences where an ASO could block splicing machinery and prevent exclusion of exon 2 in the final transcript, leading to increased GAA expression.^55^, ^56^ Goina et al. (2017) performed a similar exon 2 splicing modulation with an ASO and successfully reduced glycogen accumulation in patient‐derived myotubes.^57^ Other ASO studies have sought to reduce glycogen by interfering with GYS1 expression. Clayton et al. (2014) demonstrated that treating Pompe mice with a phosphorodiamidate morpholino oligonucleotide (PMO) designed to induce exon 6 skipping in the murine *Gys1* mRNA resulted in the degradation of messenger RNA due to the frame shift variant.^16^ However, to facilitate PMO delivery to muscle via systemic administration, the PMO was conjugated with a cell penetrating peptide (GS‐PPMO), and the development of PPMO drugs for human use is significantly challenged by their potential toxicity, as observed in primate studies.^47^ This toxicity is likely due to the cationic nature of the peptide at the doses required for efficacious exon skipping in the primate musculature.^16^, ^58^

Benefiting from advancements in ASO technology, we identified a potent mouse specific ASO that safely knocks down mouse *Gys1* expression by Rnase H1 mechanism in vivo. Using both prevention and reversal treatment modalities, we demonstrated the potential of the *Gys1* ASO approach to serve as efficacious SRT in the *Gaa^−/−^* mouse model of Pompe disease.

The *Gaa^−/−^* Pompe mice start to accumulate glycogen in skeletal muscle as early as 3 weeks of age but develop a progressive statistically significant defect in motor functions from 6 months of age. We have attempted to address the importance of timing in two separate cohorts, starting the ASO treatment at 1‐ and 3‐month‐old mice respectively. Taken together, our data indicates that long‐term elimination of muscle glycogen synthesis leads to a significant reduction of glycogen accumulation and autophagic buildup, as well as a tendency towards functional improvement in severely affected older Pompe mice.

There was a tendency for different ED50 values between ASO#1 and ASO#2 (24.2 vs. 45.2 mg/kg, Figure S1A). These values were determined in wild type mice, where ASO#1 and ASO#2 were administered twice a week for 5.5 weeks at total weekly doses of 12.5, 25, 50, and 100 mg/kg. In contrast, in young *Gaa* ^−/−^ mice treated with ASO#1 and ASO#2 at 25 mg/kg once a week for 16 weeks (Figure 2B and C), the differences in efficacy between the two ASOs were minimal. The extended treatment duration, along with the differences in disease state between *Gaa* ^−/−^ and wild‐type mice likely resulted in nearly complete suppression of *Gys1* mRNA and undetectable protein levels for both ASOs.

Interestingly, we observed superior activity of *Gys1* ASO in the skeletal muscle of Pompe mice compared to wild type, healthy mice (Figure S1B–D). We hypothesise that this phenomenon may be the result of increased permeability to ASO of the damaged muscle combined with facilitated escape of the ASO from defective endosomal compartments upon cellular uptake.

Despite the positive results obtained by knocking down *Gys1* expression with ASOs, a residual amount of glycogen remained in the skeletal muscle of 8‐month‐old *Gaa^−/−^* mice. This may be attributed to compensatory mechanisms, or the presence of very low levels of residual GYS1 enzymatic activity.

In the heart, we observed a variable degree of GYS1 protein reduction. On average, ASO#2‐mediated Gys1 knockdown was more effective in older mice compared to younger mice (73% in 1‐month‐old Gaa^−/−^ vs. 91% in 4‐month‐old Gaa^−/−^; Table S1). This difference may be attributed to disease progression, as older Pompe mice exhibit more advanced pathology, including increased lysosomal permeability and membrane disruption, which could enhance ASO uptake and activity. In contrast to skeletal muscle, the impact of ASOs on glycogen reduction in the heart was limited. This could be due to distinct biochemical processes or functional pathway in the heart making it less responsive to GYS1 knockdown by ASOs. The pathophysiology in the heart may be more complex or involve other factors beyond glycogen accumulation that limit the therapeutic efficacy of the ASO. To further improve the efficacy of our *Gys1* ASO‐based SRT approach both in the skeletal muscle and heart, we combined the ASO treatment with ERT. Our data shows that the combination of *Gys1* ASO with rhGAA results in enhanced reduction of glycogen content and reversal of the autophagy defect in the skeletal muscle of old *Gaa^−/−^* mice. The molecular changes induced by the ASO and ERT treatment also led to the preservation of motor function in the aging Pompe mice.

As expected, we demonstrated that cardiac muscle responded better to ERT compared to skeletal muscle. While the impact of ASO#2 on heart glycogen levels was negligible in the 1‐month‐old cohort, we observed a tendency to decrease cardiac glycogen in the older 4‐month‐old cohort. Although modest, this diminution may have contributed to the observed improvement of the pathology in Pompe mice. Furthermore, when *Gys1* ASO#2 was used in combination with ERT, a more substantial reduction in autophagic markers was archived, facilitating enhanced clearance of both glycogen and autophagic debris, thereby promoting cellular homeostasis in cardiac muscle. Notably, this combined approach was particularly effective in skeletal muscle, a tissue that typically less responsive to ERT alone.

Although ERT alone showed benefit in reducing centralised nuclei, the combination of *Gys1* ASO#2 and ERT led to a more pronounced reduction in centralised nuclei in comparison to the control ASO group. This highlights the advantage of targeting multiple pathophysiological pathways simultaneously to enhance muscle regeneration and repair. The combination of Gys1 ASO#2 and ERT may work synergistically to improve cellular homeostasis and promote more effective muscle recovery, particularly in tissues affected by glycogen accumulation and autophagic dysfunction, such as skeletal muscle in Pompe disease. These findings highlight the potential of combining *Gys1* ASO with ERT as a promising therapeutic strategy for Pompe disease, overcoming tissue‐specific limitations and supporting overall cellular function.

Ullman et al. (2024)^32^ recently reported their preclinical studies of MZ‐101, a selective and noncompetitive inhibitor of GYS1 which reduced glycogen accumulation, similar to that of ERT alone in Pompe mouse models. Similar to our studies they found that MZ‐101 when utilised in combination with ERT had an additive effect of normalising glycogen concentrations in the muscles. These data suggest that substrate reduction therapy with GYS1 inhibition may be a promising therapeutic approach for Pompe disease. Of note, they found 60% reduction in the glycogen stores in the cardiac muscle. We do not see such a significant reduction in glycogen in cardiac muscle perhaps because the treatments involve two different molecules and mechanisms. We would like to emphasise that ERT treats cardiac muscle very efficiently and there is no need for additional treatment of the heart with ASOs. The problem is with skeletal muscle where ERT has limited efficiency, and adjunct therapy of ERT and ASOs provide a significant benefit. Recently, Maze Therapeutics announced reductions in blood mononuclear cell glycogen across dose levels 10 days after administration from a Phase I clinical trial of the small molecule GYS1 inhibitor MZE001 in healthy individuals (ClinicalTrials.gov Identifier: NCT05249621). The results from the clinical trial in patients with Pompe disease are pending. There is a great need for trials of potential molecules that will enhance delivery in skeletal muscle to reduce the progressive glycogen and autophagic build‐up. It is expected that GYS1 ASOs have the potential to be used as an alternative treatment approach, as monotherapy in LOPD with little or no cardiac involvement, or in combination with ERT primarily for IOPD in future clinical trials for patients with Pompe disease.

## 4 MATERIALS AND METHODS

### 4.1 Identification of antisense oligonucleotides (ASOs) targeting mouse *Gys1*

All ASOs used in the studies described here were 16 nucleotides in length with a phosphorothioate (PS) backbone and three 2′‐constrained ethyl (cEt)‐modified nucleotides at both ends (3‐10‐3 gapmer configuration). A well‐characterised control ASO which does not hybridise to any mouse mRNA sequence was included in the experiments. The oligonucleotides were synthesised and purified as previously described.^59^ ASOs targeting murine *Gys1* were designed and initially screened for activity in B16‐F10 cells via electroporation. The most active ASOs were subsequently evaluated in wild‐type mice to identify ASOs that were safe and active in vivo. A dose–response confirmation experiment for the two lead ASOs, ASO#1 and ASO#2, was performed in wild‐type mice by subcutaneous injection twice a week for 5.5 weeks at 12.5, 25, 50, and 100 mg/kg total weekly doses (shown in Figure S1A). All ASOs were formulated in phosphate buffered saline (PBS) and were administered to mice by subcutaneous injection. The mouse ASO screening studies were conducted under protocols approved by the Institutional Animal Care and Use Committees at Ionis Pharmaceuticals.

### 4.2 Characterisation of the *Gaa^−/−^* animal model of Pompe disease

The *Gaa^−/−^* Pompe mouse model was a gift of Dr. Nina Raben.^60^ The mouse model is a constitutive homozygote knockout of the murine acid alpha‐glucosidase (*Gaa*) gene. One‐month‐old *Gaa^−/−^* mice, which show only the initial signs of tissue glycogen accumulation without severe myopathy, were used in prevention studies, whereas 3‐month‐old *Gaa^−/−^* mice, which show marked glycogen accumulation and motor function impairment, were used in reversal studies. All the intervention studies performed in either the *Gaa^−/−^* mouse model or wild‐type C57BL/6 control mice were conducted in accordance with the Institutional Animal Care and Use Committee at the University of California, Irvine (UCI), under protocol IACUC AUP‐19‐075, and were consistent with Federal guidelines. The mice were housed at UCI animal facility that maintained a constant temperature (22°C) and controlled light and dark cycles (12:12). The overall condition of the mice was monitored daily throughout the experimental process to assess general health and mitigate any pain and suffering.

### 4.3 Administration of ASOs to *Gaa^−/−^* mice

*Gaa^−/−^* or wild‐type control mice were dosed with ASOs once a week at 25 mg/kg via subcutaneous injection. For the early‐treatment study (Cohort 1) described in Figure 2, 1‐month‐old Gaa^−/−^ mice were randomly divided into groups for treatment with different *Gys1* ASOs, control ASO, or vehicle (PBS), *n* = 12–17 mice per group. To study therapeutic effects of *Gys1* ASO in mice with established pathology (Cohort 2) described in Figure 3, 3‐month‐old *Gaa^−/−^* mice were randomly divided into three groups for treatment with *Gys1* ASO#2 (*n* = 15), control ASO (*n* = 10), or vehicle (PBS) (*n* = 10). The mice received a total of 16 weekly ASO doses (16 weeks duration) and were sacrificed 48 h after the last ASO dose. Body weight changes, and the weight of liver, kidney, and spleen were recorded to exclude potential toxicity of the ASOs in the context of the long dosing paradigm in diseased *Gaa^−/−^* mice.

### 4.4 Adjunct treatment using *Gys1* ASO in combination with ERT in *Gaa^−/−^* mice

Four‐month‐old *Gaa^−/−^* mice were dosed with *Gys1* ASO#2 or control ASO by subcutaneous injection once a week at 25 mg/kg for 6 weeks. Starting from week 7 the mice additionally received 20 mg/kg recombinant human GAA (rhGAA) (Genzyme Corporation, Cambridge, MA; Cat.: NDC 58468‐0160‐1) via intravenous injection once every 2 weeks. The antihistaminic agent diphenhydramine hydrochloride was administered to the mice via intraperitoneal injection 15 min before receiving the human enzyme, starting from the second rhGAA administration. The weekly subcutaneous ASO administration continued throughout the ERT phase for a total of 16 doses. The mice were euthanised at 8 months of age, after having been treated with *Gys1* ASO for 4 months, the last two of which overlapped with the ERT; *n* = 5 per treatment group.

### 4.5 Accelerating rotarod performance test

Muscle function was evaluated using a rotarod apparatus (Rotamex‐5, Columbus Instruments, Columbus, OH). Briefly, mice were placed on a rotarod whose speed was set to progressively increase from 4 to 40 rotations per minute (rpm) in 300 s. The latency to fall from the rotating rod was recorded as a measure of muscle strength. Assessments were done on three consecutive days, three to five trials each day with at least 2‐min rest intervals between the trials. The mean latency time to fall off the rotarod from three independent trials from the last two days was used for analysis.

### 4.6 Biochemical analysis of serum from mice for toxicity studies

Blood samples were collected from mice via cardiac puncture under deep anesthesia using isoflurane inhalation. Immediately after collection, approximately 300–500 µL of whole blood were placed into serum separator tubes (Greiner Bio‐One Minicollect tubes; Cat.: 450472). Samples were allowed to clot for at least 20 min at room temperature and then centrifuged at 3000 rpm for 15 min. Serum was transferred to fresh tubes and stored at –80°C before being used for clinical biochemistry evaluation at IDEXX Laboratories (IDEXX Laboratories Inc., Maine, USA). Parameters measured include alanine aminotransferase, aspartate aminotransferase, alkaline phosphatase, blood urea nitrogen, creatine kinase.

### 4.7 Gene expression analysis using quantitative real‐time PCR

Tissue samples were isolated from mice and snap frozen in liquid nitrogen. Frozen tissues were homogenised in TRIzol reagent (Thermo Scientific, Waltham, MA; Cat.: 15596026), and total RNA was extracted according to the manufacturer's instructions. Real‐time qPCR analysis was performed using the following reagents: mouse *Gys1* (Taqman™ assay ID: Mm01962575_s1), mouse Gapdh (Taqman™ assay ID: Mm 99999915_g1, housekeeping gene). In the initial assessment of ASO activity in wild‐type mice (Figure S1A) real‐time qPCR analysis was performed using the following primer‐probe sets: mouse *Gys1* (forward primer: 5′‐TGATGAAGAGAGCCATCTTTGC‐3′; reverse primer: 5′‐AGGAGTCGTCCAGCATGTTGT‐3′; probe: 5′‐ACTCAGCGGCAGTCTTTCCCACCA‐3′), mouse cyclophilin A (forward primer: 5′‐TCGCCGCTTGCTGCA‐3′; reverse primer: 5′‐ATCGGCCGTGATGTCGA‐3′; probe: 5′‐CCATGGTCAACCCCACCGTGTTC‐3′). Reactions were conducted in triplicate on a QuantStudio7 real‐time PCR system (Thermo Fisher Scientific, Waltham, MA), following the manufacturer's instructions.

### 4.8 Biochemical measurement of tissue glycogen content

Mouse tissues were collected, snap frozen in liquid nitrogen upon collection, and stored at –80°C. Approximately 10 mg of frozen quadriceps muscle were rinsed in 300 µL of ice‐cold PBS, then homogenised in 200 µL of double‐distilled water using a Wheaton dounce tissue grinder (DWK Life Sciences, Rockwood, TN) as follows: 10–20 passes on loose pestle followed by 10–20 passes on tight pestle, keeping the tissue grinder on ice. The tissue lysates were then heated at 100°C for 10 min and then centrifuged at 15 000 × *g* for 10 min at 4°C before glycogen measurement. Glycogen concentration was measured using a colorimetric assay (Abcam, Ab65620), according to the manufacturer's instructions. The resulting signals were analysed using the BioTek Gen5 software (Agilent Technologies, La Jolla, CA), and normalised to the mount of the tissue sample used.

### 4.9 Western blot analysis of GYS1 protein and autophagy markers

Approximately 30 mg of frozen tissue was homogenised in 500 µL of RIPA buffer (Sigma‐Aldrich, St. Louis, MO; Cat.: R0278) supplemented with a proteinase inhibitor cocktail (Sigma‐Aldrich, St. Louis, MO; Cat.: P8340) using a handheld electric homogeniser (Qsonica, Newtown, CT; Cat.: XL‐2000). The tissue homogenate was then agitated on a Benchmark Roto‐Mini Plus (Benchmark Scientific, Sayreville, NJ) for 2 h at 4°C. Lastly, the tissue lysate was centrifuged at 20 000 × *g* at 4°C for 30 min, and the supernatant was collected for analysis. The sample protein concentration was measured using the Micro BCA protein assay kit (Thermo Fisher Scientific, Waltham, MA; Cat.: 23235). 20 µg of total protein lysate was resolved by electrophoresis on Bis‐Tris 4–12% NuPAGE polyacrylamide gels using the Novex Mini Cell system (Invitrogen/Thermo Fisher Scientific, Waltham, MA). The proteins were then transferred to a PVDF membrane, which was later blocked with 1% bovine serum albumin (BSA) in Tris‐buffered saline containing 0.1% Tween 20 (TBS‐T). The primary antibodies used in our studies were: anti‐GYS1 (Abcam, Waltham, MA; Cat.: ab 40810; 1:5 000 dilution), anti‐LC3B‐I/II (Abcam, Waltham, MA; Cat.: ab192890; 1:5000 dilution), anti‐p62 (Abcam, Waltham, MA; Cat.: ab56416; 1:20 000 dilution), anti‐LAMP1 (Abcam, Waltham, MA; Cat.: ab27170; 1:2000 dilution), anti‐GAPDH (Abcam, Waltham, MA; Cat.: ab18160; 1:10 000 dilution; served as loading control), anti‐β‐ACTIN (Abcam, Waltham, MA; Cat.: ab8227; 1:5000 dilution; served as alternative loading control). Horseradish peroxidase (HRP)‐linked goat anti‐mouse or goat anti‐rabbit secondary antibodies were used (1:5000 dilution). On average, each experiment included *n* = 5 samples per group, with three technical replicates per sample. Densitometric quantification was performed using ImageJ software (National Institutes of Health, Bethesda, MD).

### 4.10 Periodic acid‐Schiff histological analysis

Periodic acid‐Schiff (PAS) staining was performed to detect glycogen in histological tissue sections. Briefly, the quadriceps muscle was dissected and fixed in a 4% neutral buffered formalin solution at room temperature for 20 h. The fixed tissue was then rinsed in PBS and transferred to a 70% ethanol solution for storage. Paraffin embedding, sectioning (5 µm), and PAS staining were performed by Histoserv (Germantown, MD).^61^ Images of the histological slices were captured using an Aperio AT2 scanner (Leica Biosystems, Germany).

PAS Staining Signal Intensity Quantification: The fuchsia‐coloured intensity within muscle fibres was measured using AI Microscopy Image Analysis Software (Imaris 10.2) in three steps: (1) Image conversion: 20× images from the Leica microscope were converted to.ims files using the Imaris file converter, then to greyscale for easier processing. The voxel size was adjusted according to the microscope manual. (2) Image segmentation: Machine learning segmentation was applied to distinguish foreground and background, followed by manual review to ensure accurate cell segmentation. (3) Data exporting and the median intensity was used for group analysis. For each treatment and control group, five images of quadriceps muscle from 4–5 animals, with at least 200 cells per image were analysed. To ensure accuracy, 50% of the images were reanalysed by a different individual. To quantify the number of myofibres with centralised nuclei, a minimum of 600 myofibres per animal were manually evaluated in the PAS‐stained sections of quadriceps muscle (*n* = 4–5 mice per each group). The data is presented as the percentage of myofibres with a centralised nucleus.

### 4.11 Immunohistochemistry (IHC) studies

For lysosomal analysis, the expression of lysosomal associated membrane protein 1 (LAMP1) was assessed using IHC in tissue sections of quadriceps muscle embedded in frozen section medium (Fisher Scientific, Hampton, NH; Cat.: 22‐046‐511), as described previously.^62^ Briefly, 8 µm‐thick cryosections (10 sections per each animal, 5 animals per treatment group) were fixed in 4% paraformaldehyde (PFA), blocked in donkey serum, and stained with an anti‐LAMP1 primary antibody (Abcam, Waltham, MA; Cat.: ab24170; 1:500 dilution) at 4°C overnight. The sections were then washed three times with PBS and incubated with fluorescein‐conjugated secondary antibody (Thermo Fisher Scientific, Waltham, MA; Cat.: A10042; 1:500 dilution) for 1 h at room temperature, then washed and mounted with DAPI‐containing mounting media (Vectashield, Vector Laboratories, Newark, CA; Cat.: H‐1200‐10). The slides were imaged on a Zeiss LSM 900 confocal microscope (Carl Zeiss Microscopy, White Plains, NY). For the quantification of the LAMP1 staining, the percentage of LAMP1‐positive myofibres was calculated and compared across treatment groups.

Nissl (toluidine blue) staining was used to study the morphology and pathology of neural tissue. The spinal cord was harvested from mice, fixed in 4% paraformaldehyde (PFA) for 24 h, and then incubated in a solution containing 30% sucrose in PBS overnight, embedded in optimal cutting temperature compound (OCT compound), and cryo‐sectioned. Subsequently, the 10‐micron cryosections were stained with 1% toluidine blue containing 1% borate solution as previously described.^63^, ^64^ Briefly, the slices were placed in Toluidine blue solution for 30 s and rinsed in triple distilled water. Stained slices were examined using light microscopy.

### 4.12 Statistical analysis

Statistical analysis was performed using GraphPad Prism software (GraphPad Software, Boston, MA), or RStudio software (RStudio, Boston, MA) in the case of Figure 1B. Unpaired *t*‐test, one‐way ANOVA, or two‐way ANOVA were used, as specified in the respective figure legends. One‐way ANOVA was used only when comparing between genotypes, among age groups, or among treatment groups). Contingency table analysis with Fisher exact test was used to analyse the neurons with eccentric nuclei in the cervical region of the spinal cord. *p*‐values lower than .05 were considered statistically significant; *p < .05, **p < .01, ***p < .005, and ****p < .0001, ns = not significant. Error bars indicate the standard error of the mean (SEM).

## AUTHOR CONTRIBUTIONS

Lan Weiss, Alyaa Shmara, Angela Martin, Hong Yin, Pallabi Pal, Cheng Cheng, Lac Ta, Victoria Boock, Yasamin Fazeli, Michele Carrer, Marvin Paguio, Jonathan Lee and Hong Yin performed experiments, collected, and analyzed data. Lan Weiss, Alyaa Shmara, Marvin Paguio and Jonathan Lee prepared the initial manuscript. Lan Weiss, Michele Carrer, Tamar R Grossman, Nina Raben, Paymaan Jafar‐Nejad and Virginia Kimonis contributed to the research design. Lan Weiss, Michele Carrer, Alyaa Shmara, AV and Virginia Kimonis contributed to data interpretation and manuscript editing. Michele Carrer, John Weiss, Tamar R Grossman, Nina Raben, Paymaan Jafar‐Nejad and Virginia Kimonis conceptualized and supervised the project.

## CONFLICT OF INTEREST STATEMENT

Lan Weiss, Hong Yin, Pallabi Pal, Cheng Cheng, Lac Ta, Victoria Boock, Yasamin Fazeli, Mindy Chang, Marvin Paguio, Jonathan Lee, and Howard Yu report no disclosures. Nina Raben reports no disclosures. Virginia Kimonis is the Principal Investigator for the Rare Diseases Sanofi Registry and has received funding for an investigator initiated and outreach education programs for lysosomal storage diseases. Alyaa Shmara and Angela Martin have received fellowship funding from Sanofi‐Genzyme. Michele Carrer, Paymaan Jafar‐nejad, and Tamar Grossman are current or former paid employees of Ionis Pharmaceuticals.

### ETHICS

All animal procedures were conducted in accordance with the ethical standards of the Institutional Animal Care and Use Committee (IACUC) at the University of California‐Irvine, under protocol IACUC AUP‐19‐075, and were consistent with Federal guidelines.

## Supporting information



Supporting Information

Supporting Information

Supporting Information

Supporting Information

Supporting Information

## Data Availability

The data that support the findings of this study are available from the corresponding author upon reasonable request.
