# Supplementary figures and images for: Skeletal muscle effects of antisense oligonucleotides targeting glycogen synthase 1 in a mouse model of Pompe disease
Source: Clin Transl Med. 2025 Apr 23;15(4):e70314. doi: 10.1002/ctm2.70314 (PMC12017901; doi:10.1002/ctm2.70314)

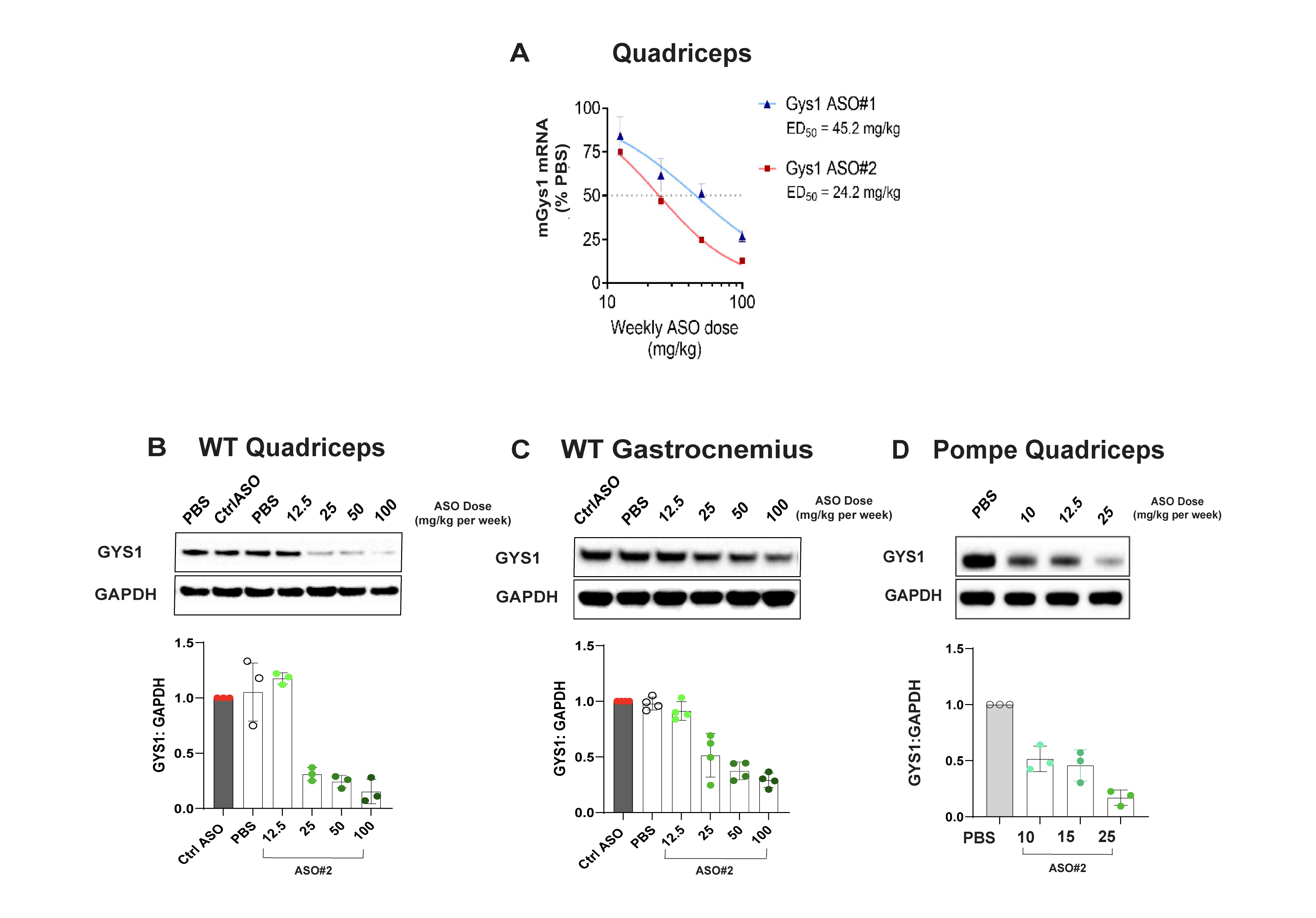

Supplement: Supplementary file 1 — Supporting Information [file CTM2-15-e70314-s001.tif]

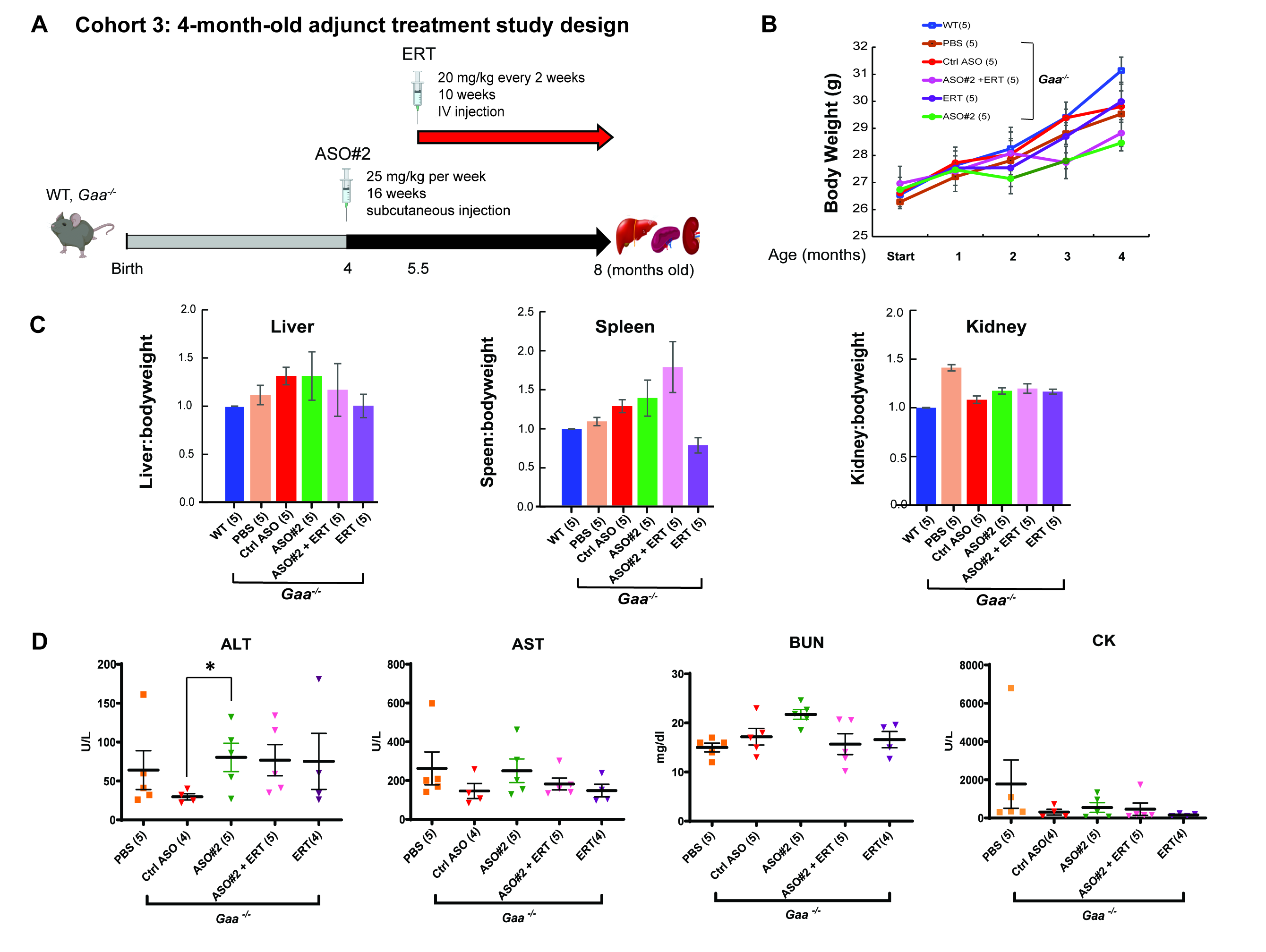

Supplement: Supplementary file 2 — Supporting Information [file CTM2-15-e70314-s002.tif]

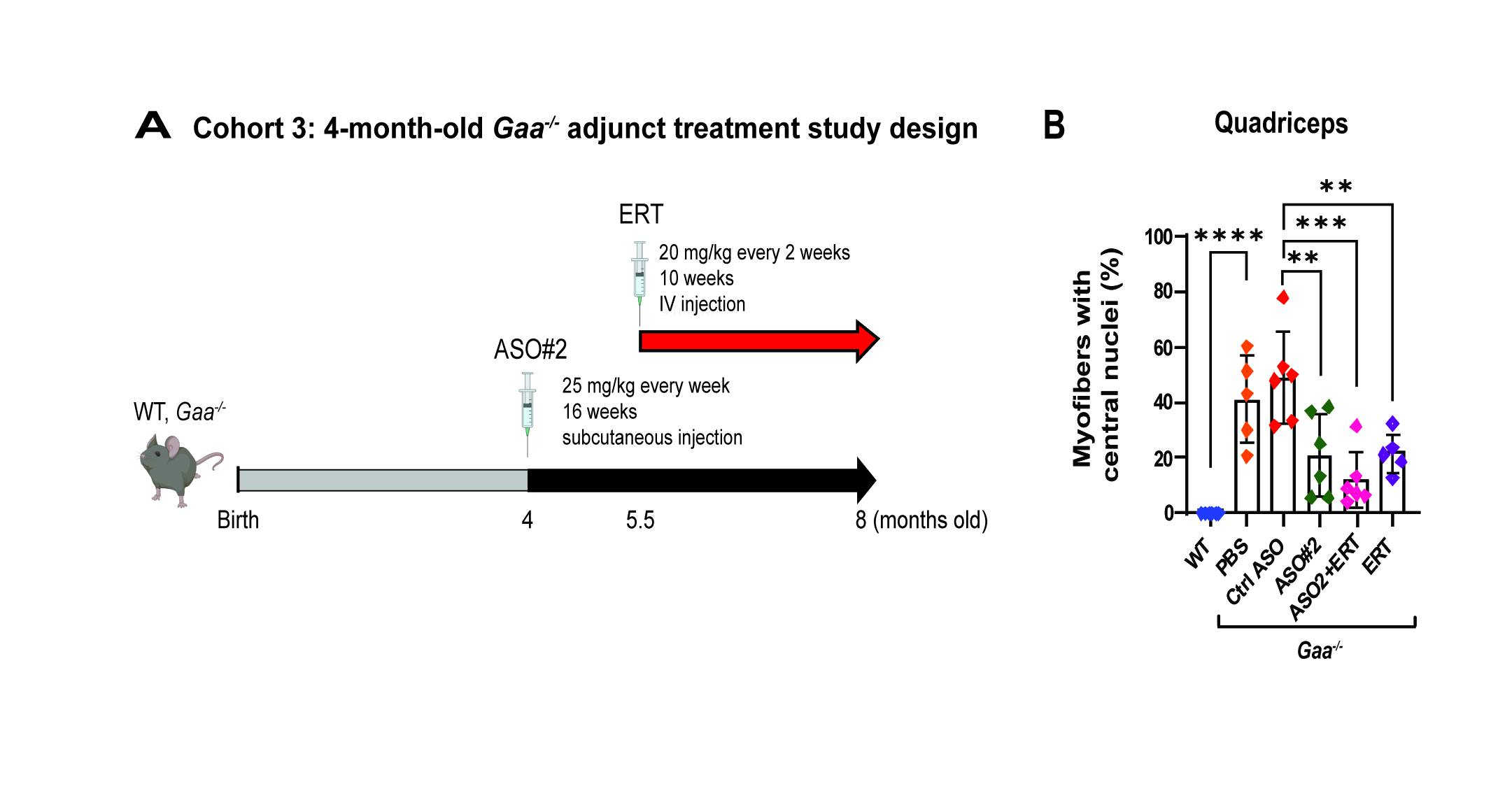

Supplement: Supplementary file 3 — Supporting Information [file CTM2-15-e70314-s003.tif]

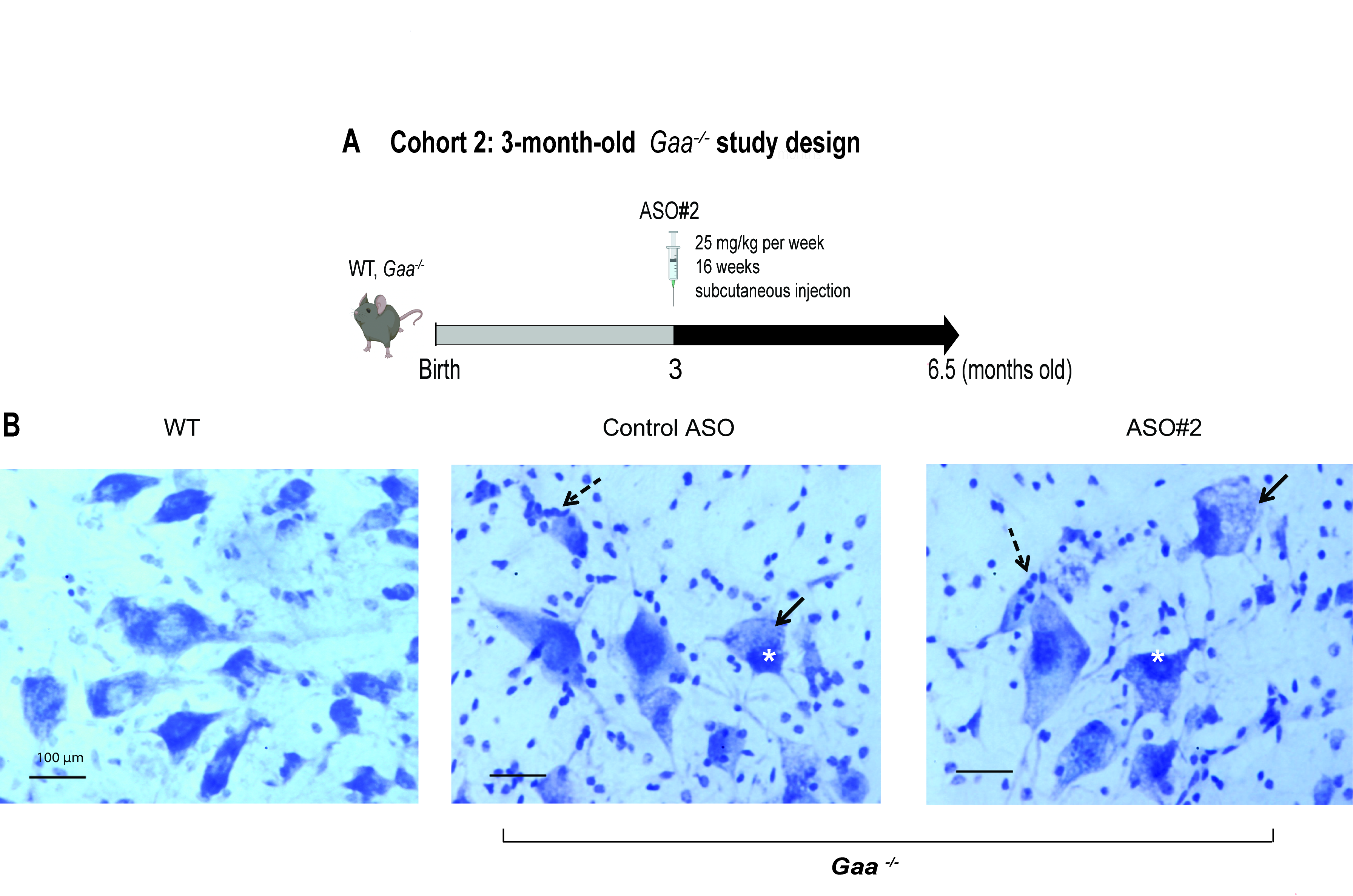

Supplement: Supplementary file 4 — Supporting Information [file CTM2-15-e70314-s005.tif]
