# Supplementary material for: Skeletal muscle effects of antisense oligonucleotides targeting glycogen synthase 1 in a mouse model of Pompe disease
Source: Clin Transl Med. 2025 Apr 23;15(4):e70314. doi: 10.1002/ctm2.70314 (PMC12017901; doi:10.1002/ctm2.70314)
Supplement: Supplementary file 5 — Supporting Information [file CTM2-15-e70314-s004.docx]

**Supplementary Table 1. Summary of percent reduction over control ASO of *Gys1* mRNA, glycogen content, and GYS1 protein in *Gaa^-/-^* mice treated with ASO, ASO+ERT, or ERT alone.**

Mice from three separate cohorts were dosed with the indicated treatment, starting at 1, 3, or 4 months of age, and ending at 4.5, 6.5, or 8 months of age, respectively. The data is reported as percent reduction of *Gys1* mRNA, GYS1 protein and glycogen content in *Gaa^-/-^* mice treated with ASO, ASO+ERT, or ERT alone, compared to control ASO- treated mice.

|  |  | **% reduction (vs. control ASO**) | | | | | |  |
| --- | --- | --- | --- | --- | --- | --- | --- | --- |
|  |  | ***Gys1* mRNA**  (±95% C.I.) | **GYS1 protein**  **(n = 5-7)** | | | **Glycogen content**  (±95% C.I.) | | |
| **Mouse cohort** | **Treatment** | **Quadriceps** | **Quadriceps** | **Diaphragm** | **Heart** | **Quadriceps** | **Diaphragm** | **Heart** |
| 1-month-old *Gaa^-/-^*  16-week treatment End: 4.5 months | ASO#1  (n = 12) | 83.64  (77.40 - 88.16) | 98 | 85 | 70 | 41.13  (63.03 - 6.28) | 15.12  (43.32 - 21.33) | 6.75  (40 - (-44.9)) |
|  | ASO#2  (n = 12) | 88.58  (83.61 - 92.06) | 100 | 98 | 73 | 58.47  (71.82 - 38.78) | 57.07  (75.70 - 24.14) | 1.61  (38.8 - (-68.9)) |
| 3-month-old Gaa^-/-^  16-week treatment End: 6.5 months | ASO#2  (n = 10) | 83.34  (89.65 – 73.21) | 100 | 80 | 70 | 32.66  (51.22 - 7.04) | 32.33  (52.83 - 2.93) | 14.4  (57.2 - 5.23) |
| 4-month-old Gaa^-/-^  16-week treatment End: 8 months | ASO#2  (n = 5) | 99.26  (99.63 - 98.51) | 100 | 90 | 91 | 40.32  (68.9 - 12.68) | 50.86  (69.93 - 19.72) | 34.55  (65.69 - 19.9) |
|  | ASO#2+ERT  (n = 5) | 93.89  (97.663 - 84.09) | 97 | 85 | 87 | 54.65  (75.11 - 17.38) | 83.13  (91.70 - 65.68) | 87.51  (97.33 - 41.59) |
|  | ERT  (n = 5) | 7.83  (47.1 - (-60.6)) | 25 | 46 | 11 | 28.57  (68.3 - (-61.1)) | 49.74  (77.32 - 10.20) | 97.72  (99.83 - 69.83) |

C.I: confidence interval
